# Fermentative Production of Fructo-Oligosaccharides Using *Aureobasidium pullulans*: Effect of Dissolved Oxygen Concentration and Fermentation Mode

**DOI:** 10.3390/molecules26133867

**Published:** 2021-06-24

**Authors:** Xinquan Liang, Chenglin Li, Weifeng Cao, Weilei Cao, Fei Shen, Yinhua Wan

**Affiliations:** 1 Department of Sugar Engineering, College of Light Industry and Food Engineering, Guangxi University, Nanning 530004, China; lxq10713@163.com (X.L.); a860835337@gmail.com (C.L.); 2 State Key Laboratory of Biochemical Engineering, Institute of Process Engineering, Chinese Academy of Sciences, Beijing 100190, China; wlcao@foxmol.com (W.C.); fshen@ipe.ac.cn (F.S.); yhwan@ipe.ac.cn (Y.W.); 3 School of Chemical Engineering, University of Chinese Academy of Sciences, Beijing 100049, China

**Keywords:** fructo-oligosaccharides, dissolved oxygen concentration, repeated-batch culture, *Aureobasidium pullulans*

## Abstract

Fructo-oligosaccharides (FOS) are prebiotics with numerous health benefits. So far, the dissolved oxygen (DO) concentration control strategy for fermentative production of FOS is still unknown. In order to improve FOS production, the effects of DO concentration and fermentation mode on FOS using *Aureobasidium pullulans* were investigated in this study. The greatest FOS production (123.2 ± 6.2 g/L), with a yield of 61.6% ± 3.0% (g FOS/g sucrose), was obtained in batch culture under high DO concentration. Furthermore, repeated-batch culture revealed that enzyme production and FOS production were not closely associated with cell growth. By keeping the DO concentration above 5% in the repeated-batch culture, a maximum FOS concentration of 548.3 ± 37.4 g/L and yield of 68.6% ± 2.6% (g FOS/g sucrose) were obtained, which were 3.45% and 11.4% times higher than those obtained in the batch culture without DO control, respectively. Additionally, the ratios of 1-fructofuranosyl nystose (GF4) and 1,1,1,1-kestohexose (GF5) were 33.8% and 23.2%, respectively, in the product of repeated-batch culture, but these compounds were not detected in batch culture. Thus, it can be concluded that the DO concentration affects not only the yield of FOS but also the composition of FOS with different degrees of polymerization, which is the key factor in the fermentative production of FOS with a high polymerization degree.

## 1. Introduction

Fructo-oligosaccharides (FOS), such as 1-kestose (GF2), nystose (GF3), 1-fructofuranosyl nystose (GF4) and 1,1,1,1-kestohexose (GF5), are small dietary fibers with low caloric value and high prebiotic effects [1,2,3,4]. In addition to being calorie-free and non-carcinogenic sweeteners, FOS have superior functional properties such as modulation of colonic microflora, improvement of the gastrointestinal physiology and immune functions, bioavailability of minerals, metabolism of lipids and prevention of colonic carcinogenesis [4,5]. Currently, FOS are being considered as natural food ingredients in most countries because of their multiple benefits for human and animal health.

FOS are biosynthetically produced using a one- or two-stage process. In the two-stage process, the enzyme with transfructosylation activity is first produced from fungi, such as *Aureobasidium* spp. [6,7,8,9], *Aspergillus* spp. [10,11], *Bacillus subtilis* [12] and *Penicillium* spp. [13]. The FOS are then produced under controlled conditions with the extracted enzymes using sucrose as the substrate [14,15,16]. During the one-stage process, FOS are biosynthesized from fungi in bioreactors using either immobilized or free whole cells as biocatalysts [4,6,17,18,19]. The fermentative production of FOS using the one-stage process is advantageous when compared with the two-stage process because the step of purification of FOS-producing enzyme from cell extracts can be eliminated. For example, when using *A. pullulans strain* (FRR 5284) as the whole-cell biocatalyst for production of FOS, a FOS yield of 61% from 50% (*w*/*v*) sucrose was obtained [7]. When using a carbon catabolite repressor (CREA) gene disruptant *A. pullulans* D28 as the whole cells for FOS production [9], 0.58 g of FOSs/g of molasses sugar was synthesized from 350 g/L cane molasses sugar within 4 h. Nevertheless, the FOS yield when using the one-stage process is influenced by factors such as temperature, aeration rate, partial pressure of oxygen, and stirring speed [4,13,19,20]. During the fermentation production of FOS using whole cells (one-stage process) from *A. pullulans*, the FOS yield was found to reach 64.1 ± 0.0 g FOS/g sucrose when the stirring speed and temperature were 385 rpm and 32 °C, respectively, regardless of the aeration rate and partial pressure of oxygen [4]. Moreover, a macroscopic dynamic model of the production of FOS by *A. pullulans* based on sets of experimental data collected from batch and fed-batch cultures was derived [8]. A FOS concentration of 123 g/L was obtained after 40 h using Pontryagin’s method and 122 g/L using the non-linear programming method [8]. The dissolved oxygen (DO) concentration, which is influenced by the stirring speed, aeration rate, partial pressure of oxygen, and temperature, is an influencing factor in the fermentative synthesis of metabolite products by fungi that has been found to have a significant effect on *Aureobasidium* spp. [21,22] and *Aspergillus* spp. [23]. However, strategies for controlling DO during the fermentative production of FOS have not been discussed in any literature to date. Therefore, it is necessary to better characterize the role of DO during one-stage production of FOS.

In a fed-batch bioreactor, a high concentration of FOS was achieved by optimally controlling the FOS production [18]. In addition, β-fructofuranosidase was effectively produced with immobilized *A. japonicus* using repeated batch culture [24,25]. However, it was not reported whether the DO concentration should be controlled or not in the fed-batch bioreactor. Therefore, we speculate first that the FOS production maybe improved by adjusting DO concentration in a suitable fermentation mode. Furthermore, we conducted an in-depth investigation of the effects of DO concentration on FOS production. We then evaluated FOS production conducted using different fermentation modes.

## 2. Results and Discussion

### 2.1. Effect of Dissolved Oxygen (DO) Concentration on Fructo-Oligosaccharides (FOS) Production

#### 2.1.1. Effect of Stirring Speed on FOS Production

**Figure 1:**
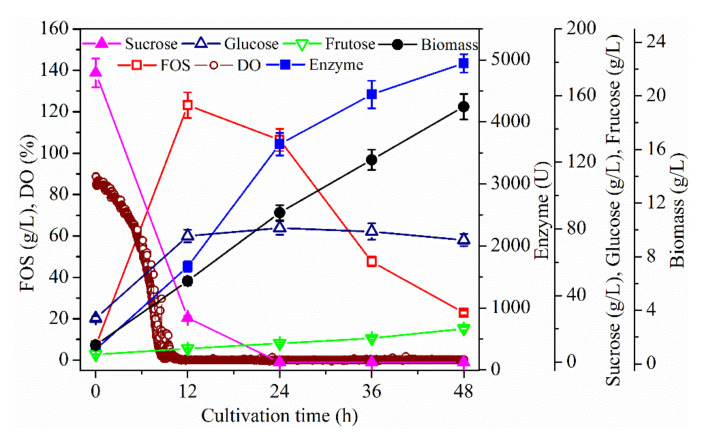
Time profiles of fructo-oligosaccharides (FOS) production in a 2.7 L bioreactor. The *A. pullulans* ipe-3 strain was cultivated in a 2.7 L bioreactor at pH 5.5, aeration rate 1.8 L/min, 25 °C, and a stirring speed of 600 rpm. The working volume was 1.8 L. Data are given as the mean ± standard deviation (SD), *n* = 2.

The DO value varied with stirring speed during fermentation using *A. pullulans* ipe-1 [21]. Gibbs and Seviour [22] also reported that the hyphae of *A. pullulans* did not show any evidence of ill effects in response to high shear rates (i.e., 750 rpm and above), even with exposure to up to 1250 rpm, suggesting that *A. pullulans* can tolerate high stirring speeds. Therefore, in order to explore the effect of stirring speed on DO and FOS production, a batch culture using *A. pullulans* ipe-3 cultivated in a 2.7-L bioreactor was first performed under the following conditions: pH, 5.5; aeration rate, 1.8 L/min; temperature, 25 °C; stirring speed, 600 rpm (Figure 1). Figure 1 shows that the FOS were mainly produced in the first 12 h, during which time the sucrose concentration decreased sharply. The DO concentration was initially 100%, then gradually fell to zero. When cells grew quickly and their concentrations were high, the detected DO value was near zero. After culturing for 12 h, the FOS concentration decreased until the end of fermentation; however, the DO concentration fell to near zero, while the cell growth, enzyme biosynthesis and fructose release increased until the end of fermentation. After the sucrose depletion, the fructose release increased more quickly. During fermentation, the glucose release increased sharply in the first 12 h due to the biosynthesis of FOS, while its concentration remained almost constant until the end of fermentation. In fact, even though *A. pullulans* could use a wide variety of carbohydrate substrates for cell growth, sucrose and glucose were found to be the best nutrients [22,23,24,25,26,27]. Thus, the continuous release of fructose was mainly facilitated by hydrolysis of the synthesized FOS by the produced enzyme. Moreover, the glucose released during the enzyme catalysis was assimilated by the strain for cell growth and enzyme production, which resulted in an almost constant concentration of glucose in the broth. Furthermore, the enzyme production was not coupled to cell growth (Figure 1), which was in accordance with the results reported by Hayashi et al. [28] and in contrast to those reported by Shin et al. [29]. However, the reason for the difference in observations was the different strains used. Thus, the DO and substrate (i.e., sucrose) concentrations were the two main factors influencing FOS production; therefore, these are discussed further in the following sections.

**Figure 2:**
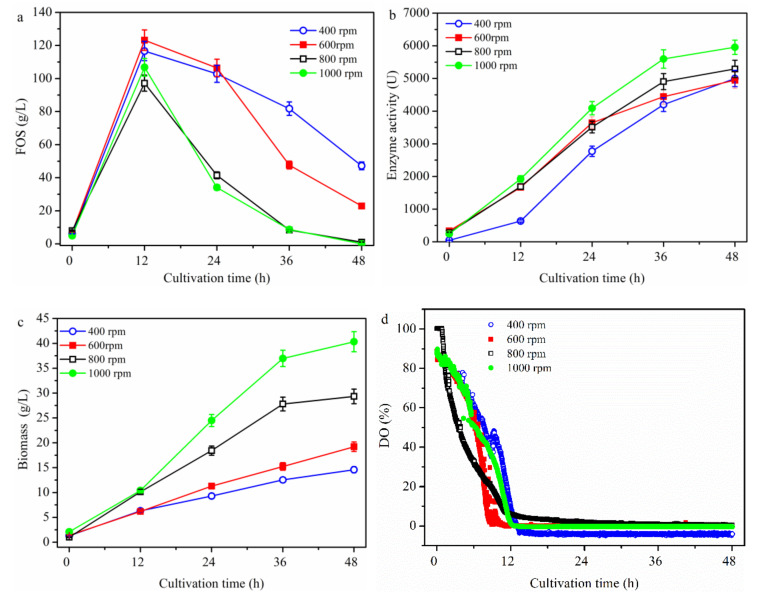
Time profiles of the batch cultivation at different stirring speed values. The *A. pullulans* ipe-3 strain was cultivated in a 2.7 L bioreactor at pH 5.5, aeration rate 1.8 L/min, and 25 °C at different stirring speeds. The time courses refer to (**a**) FOS, (**b**) enzyme activity, (**c**) biomass, and (**d**) DO. The working volume was 1.8 L. Data are given as the mean ± SD, *n* = 2.

Moreover, to further investigate the effects of stirring speed on FOS biosynthesis, batch cultures were performed with different constant stirring speeds (i.e., 400, 600, 800, and 1000 rpm). As shown in Figure 2a, the variations in FOS production were similar to those shown in Figure 1, with the maximum concentration values of FOS of 116.6 ± 5.8, 123.2 ± 6.2, 97.2 ± 4.9, and 106.8 ± 5.3 g/L being at 400, 600, 800 and 1000 rpm, respectively, after 12 h. The maximum yield of 61.6% ± 3.0% (g FOS/g sucrose) was obtained at 600 rpm (Figure 2a). The enzyme activities were 636.5 ± 31.8, 1666.3 ± 83.3, 1691.6 ± 84.6, and 1927.2 ± 96.4 U being observed at 400, 600, 800 and 1000 rpm, respectively, at 12 h (Figure 2b). After 12 h, the enzyme activity gradually increased until the end of fermentation (Figure 2b). Moreover, the enzyme activity increased sharply when the stirring speed was increased above 400 rpm, while it increased only slightly when the stirring speed was increased above 600 rpm. The biomass values increased gradually toward the end of fermentation (Figure 2c); at 12 h, the biomass was 6.3 ± 0.30, 6.2 ± 0.31, 10.2 ± 0.51, and 10.4 ± 0.50 g/L being observed at 400, 600, 800, and 1000 rpm, respectively (Figure 2b). When the stirring speed was above 600 rpm, the biomass increased sharply, while there was not much of a difference between 400 and 600 rpm or between 800 and 1000 rpm at 12 h. The reason was that the enzyme concentration was higher at 1000 rpm than that at 800 rpm under the same substrate (i.e., sucrose) concentration. Under the enough sucrose concentration before 12 h, more FOS was synthesized since the enzymatic reaction rate was higher than cell growth rate. Thus, it resulted that the biomass was not very different between 800 and 1000 rpm at 12 h. However, after 12 h, the sucrose concentration was low, and the hydrolytic activity of the produced enzyme stood out. Thus, the FOS decreased quickly, while enzyme production and cell growth increased fast. Meanwhile, the same phenomenon appeared at 400 and 600 rpm. Thus, the stirring speed showed different effects on FOS production, enzyme production and cell growth. In addition, the fructosyltransferase specific activity (Y_e/b_) at 12 h was 101.1 ± 4.2, 268.6 ± 14.1, 165.8 ± 7.6, 185.3 ± 9.1 U/(g/L biomass) being observed at 400, 600, 800, and 1000 rpm, respectively. As shown in Figure 1 and Figure 2, the FOS production was not closely associated with enzyme activity. Similarly, although cell growth and enzyme production increased with culture time, they were not coupled (Figure 2b,c). However, the DO values fell to near zero after being cultured for 12 h at all stirring speeds considered. Moreover, the FOS concentration began to decrease after culturing for 12 h. Taken together, these findings indicate that non-zero DO values (i.e., above zero) increased FOS production, while lower DO values (i.e., near zero) enhanced cell growth and enzyme production. In addition, the stirring speed intensified the dissolution of oxygen, while it did not produce gas. Cao et al. [21] and Gibbs and Seviour [22] had reported that under strictly anaerobic conditions the strain *A. pullulans* cannot grow. During the course of fermentation (cells in the exponential growth phase), when the air supply and stirring speed were completely shut off for a period of 24 h, both cell growth and β-poly (malic acid) production ceased during the period [21]. Thus, the effect of aeration rate, which is the source of oxygen, on FOS production is discussed in the following section.

#### 2.1.2. Effect of Aeration Rate on FOS Production

**Figure 3:**
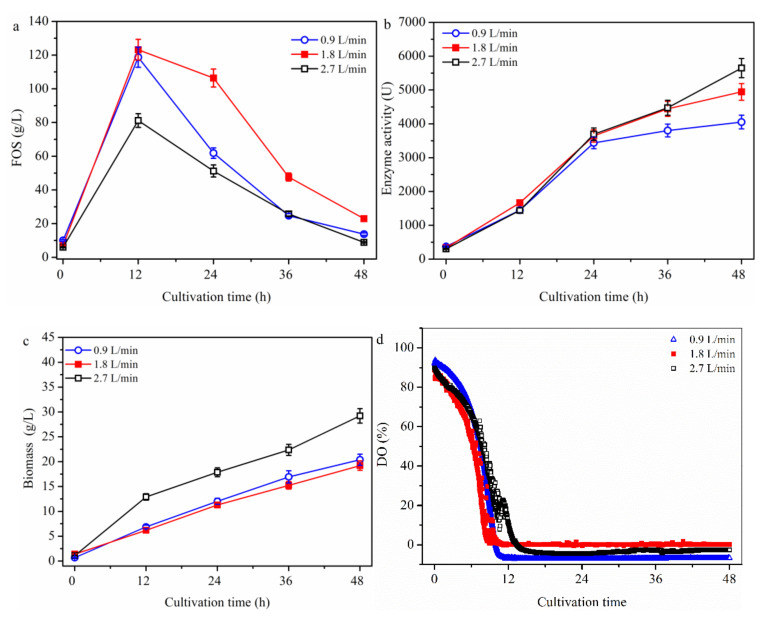
Time profiles of batch cultivation at different aeration rates on FOS production. The *A. pullulans* ipe-3 strain was cultivated in a 2.7 L bioreactor at pH 5.5 and 25 °C with different aeration rates. The time courses refer to (**a**) FOS, (**b**) enzyme activity, (**c**) biomass, and (**d**) DO. The working volume was 1.8 L. Data are given as the mean ± SD, *n* = 2.

Aeration rate was found to be a key factor in FOS production using the two stage process [30]. In order to explore the effect of aeration rate on FOS production in the one-stage process (Figure 3), a batch culture using *A. pullulans* ipe-3 cultivated in a 2.7 L bioreactor was first performed under the following conditions: pH, 5.5; temperature, 25 °C; stirring speed, 600 rpm and different aeration rates (i.e., 0.9, 1.8 and 2.7 L/min). As shown in Figure 3a, the trend in the variation of FOS production was similar to that seen in Figure 1 and Figure 2, with the maximum concentrations of FOS of 118.7 ± 5.9, 123.2 ± 6.2, and 81.2 ± 4.1 g/L at 0.9, 1.8, and 2.7 L/min being obtained at 12 h. There was not much variation in the FOS production between aeration rates of 0.9 and 1.8 L/min, while the FOS production value was much lower at 2.7 L/min than at 0.9 and 1.8 L/min. The values of enzyme production (Figure 3b) and cell growth (Figure 3c) increased steadily until the end of fermentation. The enzyme activities were 1449.6 ± 68.3, 1666.3 ± 83.3, and 1440.4 ± 72.0 U at 0.9, 1.8, and 2.7 L/min, respectively (Figure 3b). After culturing for 12 h, the enzyme activity increased slightly at 2.7 L/min. The biomass values were 6.8 ± 0.34, 6.18 ± 0.31, and 12.9 ± 0.51 g/L at 12 h at 0.9, 1.8, and 2.7 L/min, respectively (Figure 3c). In addition, the mean fructosyltransferase specific activity (Y_e/b_) at 12 h was 213.2, 269.6, and 111.7 U/(g/L biomass) being observed at 0.9, 1.8, and 2.7 L/min, respectively. Because cells grew faster at 0.9 L/min than at 1.8 L/min, there was lower enzyme production at 0.9 L/min than at 1.8 L/min after culturing for 12 h. Compared with the cell growth at different aeration rates, the rate of growth at 2.7 L/min was much higher than at 0.9 and 1.8 L/min. Thus, the higher rate of cell growth was the reason for the much lower FOS concentration and enzyme activity at 2.7 L/min. The DO concentration (Figure 3d) fell to near zero at all investigated aeration rates. This meant that the DO concentration could not be significantly improved, despite the enhanced aeration rate after culturing for 12 h, and that aeration rate affected the FOS production in the first 12 h. One of the reasons for this observation could be that the oxygen molecules were not effectively dissolved in the broth because of the low solubility of oxygen in water [31,32]. The pressurized flow of oxygen inside the reactor has also been reported to be a powerful engineering tool for intensifying the process in O_2_-dependent biochemical conversions [31]. The effects of partial pressure of the bioreactor on FOS production are discussed in the following section.

#### 2.1.3. Effect of Bioreactor Partial Pressure on FOS Production

**Figure 4:**
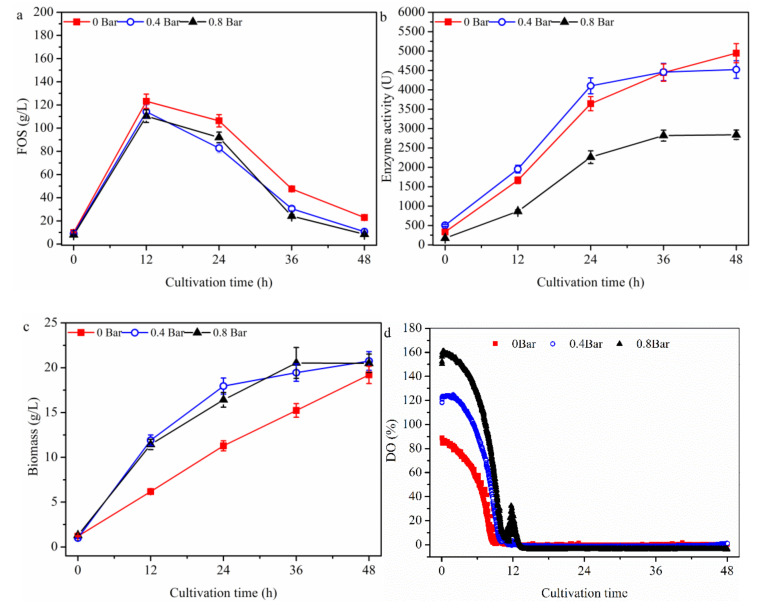
Time profiles of the batch cultivation at different partial pressures of the bioreactor. The *A. pullulans* ipe-3 strain was cultivated in a 2.7 L bioreactor at pH 5.5, aeration rate 1.8 L/min, and 25 °C with different fermenter pressures. The time courses refer to (**a**) FOS, (**b**) enzyme activity, (**c**) biomass, and (**d**) DO. The working volume was 1.8 L. Data are given as the mean ± SD, *n* = 2.

In order to explore the bioreactor partial pressure on FOS production in the one-stage process (Figure 4), a batch culture using *A. pullulans* ipe-3 cultivated in a 2.7 L bioreactor was first performed under the following conditions: pH, 5.5; temperature, 25 °C; stirring speed, 600 rpm; aeration rate, 1.8 L/min and different bioreactor partial pressures (i.e., 0, 0.4 and 0.8 bar). Enhancing the partial pressure of the bioreactor revealed that the trends in FOS production (Figure 4a), enzyme production (Figure 4b), and cell growth were similar to those shown in Figure 1, Figure 2 and Figure 3, respectively. The FOS production values (Figure 4a) were 123.2 ± 6.2, 114.2 ± 5.7, and 110.4 ± 5.5 g/L being observed at 0, 0.4 and 0.8 bar, respectively. The cell growth values (Figure 4b) were found to be significantly increased for partial pressures of less than 0.4 bar and then remain almost constant, with values of 6.2 ± 0.31, 11.9 ± 0.57, and 11.4 ± 0.61 g/L being observed at 0, 0.4, and 0.8 bar, respectively. The enzyme activities (Figure 4c) were found to increase for partial pressures of less than 0.4 bar and then to decrease, with values of 1666.3 ± 83.3, 1954.2 ± 77.7, and 863.5 ± 48.2 U being observed at 0, 0.4, and 0.8 bar, respectively. In addition, the mean fructosyltransferase specific activity (Y_e/b_) at 12 h was 268.8, 164.2, and 75.8 U/(g/L biomass) being observed at 0, 0.4, and 0.8 bar, respectively. When the partial pressure of the bioreactor was enhanced to 0.8 bar (Figure 4d), which was almost the maximum pressure used in bioreactors, the DO value significantly increased by 161.2% at the initial fermentation, but did not effectively improve after culture for 12 h when compared with the 0 bar condition. These findings indicate that the DO value could not be improved by varying only one of the factors (i.e., stirring speed, aeration rate, or partial pressure of the bioreactor). In addition, the trend of enzyme production was not in accordance with that of cell growth. For example, while the cell concentration was highest at 0.8 bar, the enzyme activity was lowest at this pressure. Thus, the metabolic pathways by which carbon source entered cell growth, FOS production and enzyme production were shifted by the varying partial pressure of the bioreactor. This occurred because the concentration of the cells, FOS, and enzyme varied under the different partial pressures of the bioreactor, although the concentration of the sucrose added was the same (Figure 4). In addition, temperature was found to greatly influence enzyme activity and oxygen solubility in the culture medium, although its effects on FOS production are not yet fully understood.

#### 2.1.4. Effect of Temperature on FOS Production

**Figure 5:**
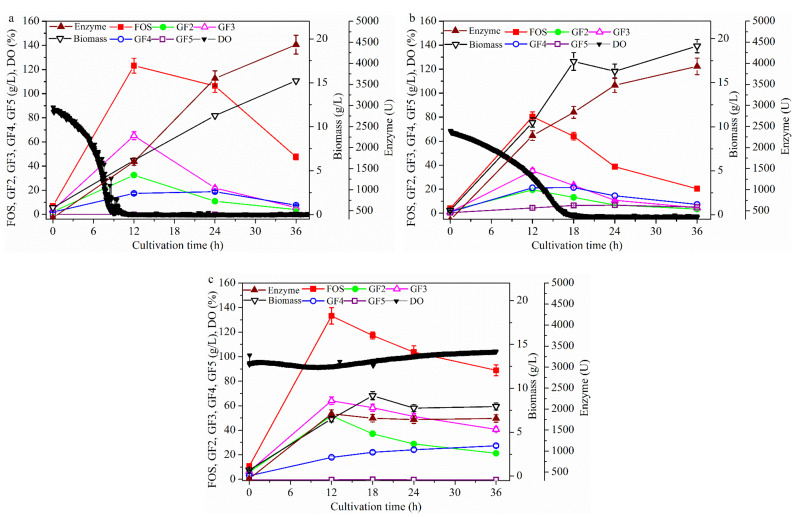
Time profiles of the batch cultivation at fermentation temperatures of 25 °C (**a**), 30 °C (**b**), and 35 °C (**c**). The *A. pullulans* ipe-3 strain was cultivated in a 2.7 L bioreactor at pH 5.5, aeration rate 1.8 L/min, and a stirring speed of 600 rpm with different fermenter pressures. The working volume was 1.8 L. Data are given as the mean ± SD, *n* = 2.

It was also reported that, while the concentration of *A. pullulans* in the cells increased as the culture temperature increased from 20 °C to 27.5 °C, an increase in the temperature beyond 27.5 °C affected cell growth negatively [26]. Similarly, Liu and Steinbüchel [33] found that temperatures higher than 25 °C during fermentation with *A. pullulans* had a negative effect on cell growth [33]. Thus, the effects of temperature on FOS production are discussed in this section. As shown in Figure 5, the trends in the variation of FOS production did not differ greatly from those under other conditions, and the maximum concentration of FOS was obtained at 12 h. When the fermentation temperature increased from 25 °C to 30 °C at 12 h (Figure 5b), the FOS concentration decreased; however, when the fermentation was further increased to 35 °C, the FOS concentration increased and the maximum concentration of 133.2 ± 6.7 g/L was obtained. This value was 8.2% higher than the concentration obtained at 25 °C. When the fermentation temperature was increased from 25 °C to 30 °C at 12 h (Figure 5c), the cell concentration increased; however, when the fermentation temperature was further increased to 35 °C, the cell concentration decreased and the maximum cell concentration of 10.5 ± 0.52 g/L was obtained at 30 °C. This concentration was 69.4% higher than the value obtained at 25 °C. Thus, cell growth showed an opposite trend to that of FOS production. At 25 °C, both cell growth and enzyme activity showed an exponential increase until the end of fermentation. At 30 °C, both cell growth and enzyme activity showed an exponential increase until 18 h, then increased slowly. However, at 35 °C, both cell growth and enzyme activity increased at the time of initial fermentation. The enzyme activity was almost constant after culturing for 12 h, while the cell concentration began to decrease after culturing for 18 h. At the end of fermentation (Figure 5c), the cell concentration (7.93 ± 0.40 g/L) and enzyme activity (1777.8 ± 88.9 U) had decreased by 58.7% and 64.0%, respectively, when compared with those at 25 °C (Figure 5a). Moreover, DO decreased slowly as temperature increased throughout the fermentation process, although it remained above 90% throughout the fermentation process at 35 °C. These findings indicate that the trend in the variation of enzyme activity was partially associated with cell growth, since only a portion of the enzyme produced by *A. pullulans* was intracellular [5,25].

Furthermore, analysis of the FOS concentration revealed that the FOS decreased rapidly when the cells grew faster after 12 h. One reason for this was that the FOS produced were mainly hydrolyzed by the enzyme produced (Figure 1) after culturing for 12 h. Another reason was that the DO value was too low after culturing for 12 h since oxygen was also required during FOS production from the enzyme [30]. However, when DO was high (Figure 5b), a high degree of polymerization of FOS was observed, although the FOS concentration was decreased. For example, the GF4 concentration increased until the end of the fermentation period at 35 °C (Figure 5c), at which time it was 2.6 times higher than that obtained at 25 °C (Figure 5a). Thus, it was speculated that the high degree of polymerization of FOS could be enhanced by keeping a high DO concentration. In addition, a high concentration of FOS could be achieved by shifting the fermentation temperature from 25 °C to 35 °C at 12 h to obtain a high cell concentration during the first 12 h and a high DO value after culturing for 12 h. These speculations are verified and discussed in the following sections.

### 2.2. Effect of Fermentation Mode on FOS Production

#### 2.2.1. FOS Production in a Repeated-Batch Culture with 500 mL Broth Discharged Each Time

**Figure 6:**
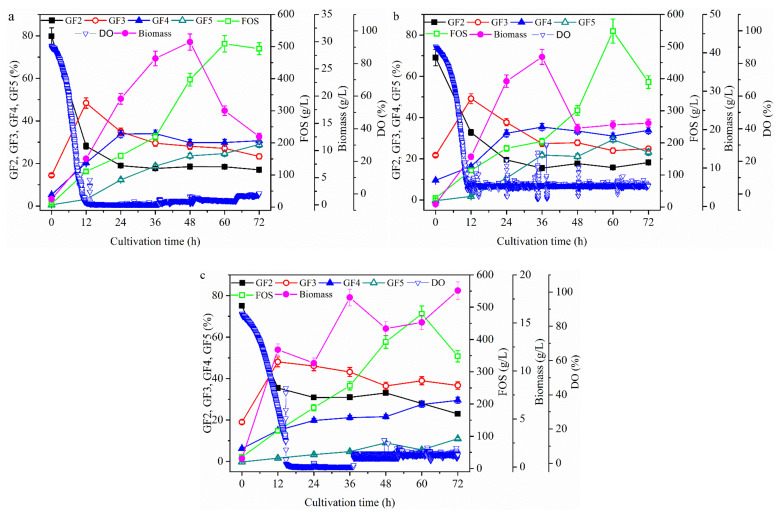
FOS production in a repeated-batch culture with 500 mL broth discharged each time. Repeated -batch cultivations at 25 °C and pH 5.5 were conducted at 600 rpm and 1.8 L/min (**a**), 5% dissolved oxygen (DO) that was cascaded to stirring speed through proportion integral differential (PID) control (**b**), and 600 rpm and 0 L/min after culturing under 600 rpm and 1.8 L/min for 12 h (**c**). For 5% DO control, the DO was allowed to automatically drop to 5% and then controlled constantly by manually adjusting the partial pressure of the bioreactor and automatically adjusting the stirring speed using the PID controller. Data are given as the mean ± SD, *n* = 2.

To explore the substrate (i.e., sucrose) on FOS production under DO control conditions, the repeated-batch culture was implemented (Figure 6a) under the same culture conditions as those shown in Figure 1 with 500 mL broth discharged each time. The FOS production steadily increased until 60 h, at which time the maximum FOS (508.8 ± 24.5 g/L) with a yield of 63.6% ± 3.1% was obtained. During this repeated-batch culture, GF5 appeared and its concentration increased steadily until the end of fermentation (72 h), when the ratio of GF5 in the FOS was 29.0%. No GF5 was detected in the batch culture. The ratios of GF2 and GF3 in FOS decreased steadily after culturing for 12 h. The ratio of GF4 in FOS increased obviously during the first 24 h, after which it remained almost constant. The DO value was then allowed to automatically drop to 5%, after which it was controlled constantly by manually adjusting the partial pressure of the bioreactor and automatically adjusting the stirring speed using a proportion integral differential (PID) controller. Another repeated-batch culture was conducted under these conditions (Figure 6b) to detect the effect of high DO value (i.e., above 5%) on FOS production. The maximum FOS concentration at 60 h was 548.3 ± 37.4 g/L, which was 7.2% higher than that seen in Figure 6a. Additionally, the ratios of GF4 and GF5 were 33.8% and 23.2%, respectively, in the product, which were not very different from those observed Figure 6a. Compared with the values in Figure 6a, the maximum FOS concentration (i.e., 548.3 ± 37.4 g/L) and yield of 68.6% ± 2.6% (g FOS/g sucrose) in Figure 6b were obtained at 60 h, which were 3.45% and 11.4% times higher, respectively. A new repeated-batch culture was then conducted under the same conditions as those shown in Figure 1 before 12 h, after which the aeration was stopped (Figure 6c) to further detect of the effect of low DO on FOS production. The maximum FOS concentration (479.6 ± 24.0 g/L) at 60 h was 5.7% lower than that shown in Figure 6a. Additionally, as shown in Figure 6c, the FOS predominantly comprised GF2 and GF3, and the ratio of GF5 was decreased by 64.5%. Taken together, these findings indicate that the DO value had little effect on the total FOS concentration after culturing for 12 h, but that it significantly influenced the composition of FOS, especially the degree of FOS polymerization in the product. Figure 6 also shows that there was a high DO value that resulted in high cell growth. The effects of DO concentration on FOS production also inferred that a high cell concentration would decrease the FOS production.

#### 2.2.2. FOS Production in a Membrane Integrated Repeated Batch Culture

**Figure 7:**
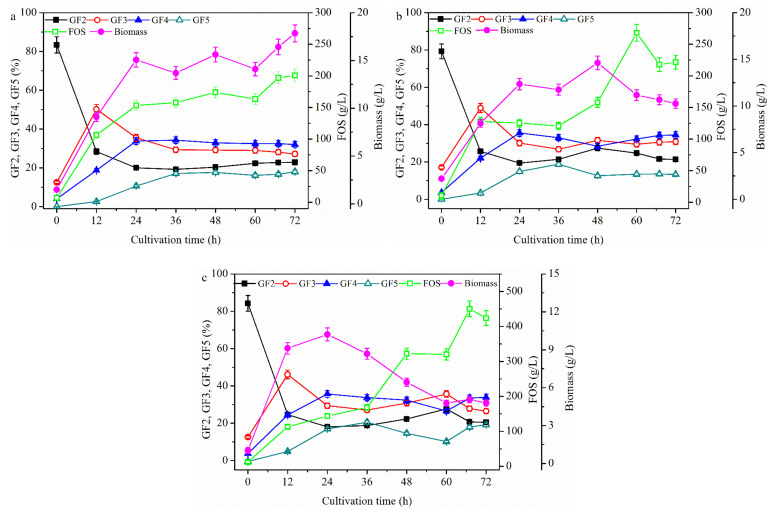
FOS production in a membrane integrated repeated batch culture. The repeated-batch cultivations at 600 rpm, aeration rate 1.8 L/min, and pH 5.5 were, respectively, carried out under 25 °C (**a**), 35 °C after culturing under 25 °C for 12 h (**b**), and 35 °C without starting the membrane after culturing under 25 °C for 12 h (**c**). The working volume was 1.8 L. Data are given as the mean ± SD, *n* = 2.

It has been reported that FOS could be effectively produced in an enzyme membrane bioreactor [34,35]. If cells are recovered with a membrane in the repeated-batch culture, it is important to determine whether FOS could be enhanced. First, FOS production was conducted in a membrane integrated repeated batch culture under the same conditions as those shown in Figure 6a and the cells in 500 mL of discharged broth were recovered using a 300 kDa membrane. As shown in Figure 7a, the FOS concentration decreased by 65.5%, and the degree of polymerization of FOS (i.e., GF5) in the product decreased by 37.8%. Surprisingly, although cells were recovered with the membrane, the final concentration of cells was not enhanced in the bioreactor. It was speculated that the cells may have been injured in the membrane system by the high shear force from crossflow filtration. As shown in Figure 5c, the cells may be used as a whole cell enzymatic system and the cell concentration is almost constant at 35 °C after culturing for 18 h. Therefore, another experiment was conducted in the same integrated fermentation under the same conditions as in Figure 7a by changing the fermentation temperature from 25 to 35 °C at 12 h. Although FOS production increased slightly under these conditions when compared to the conditions shown in Figure 7a, the production decreased by 40.4% when compared to the reaction conducted in the presence of the control without membrane filtration (i.e., the repeated-batch culture under the same conditions without a membrane system) (Figure 7c). This observation was different from those reported by Burghardt et al. [35] and Fan et al. [34], who used crude enzyme solution that did not contain any cell debris or viable cells. However, in this study, fermentation broth with viable cells was used as the crude enzyme, and the substrate and conditions met the demand of cell growth and FOS production at the same time. Thus, the results in this study were different from those obtained using crude enzyme without viable cells.

**Figure 8:**
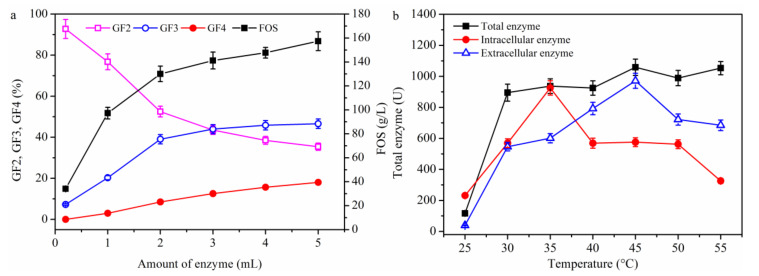
Effect of (**a**) adding enzyme and (**b**) temperature on FOS production. Enzyme indicates the fermentation broth that was used as the crude enzyme. The conditions were the same as in the enzyme activity measurement experiment but the amount of broth added was varied in (**a**), while the temperature was varied in (**b**). Data are given as the mean ± SD, *n* = 3.

The FOS production process was further studied using the broth as crude enzyme to simulate the fermentation process beyond 12 h (Figure 8a). The FOS concentration was found to increase as the enzyme volume increased in the presence of adequate substrate (i.e., sucrose), while the concentration of the low molecular weight FOS (GF2) decreased, and that of the high molecular weight FOS (GF3 and GF4) increased. These findings were similar to the results shown in Figure 6; however, GF5 was not detected. The enzyme activity from different sources was also measured (Figure 8b). The total enzyme activity increased only slightly as the temperature increased to 30 °C, at which point the whole broth was used. The intracellular enzyme activity increased below 35 °C, but started decreasing when the reaction temperature went beyond 35 °C. The activity of the extracellular enzyme increased until the temperature reached 45 °C, above which it decreased. These observations explain why the FOS production was higher in Figure 6a than in Figure 7a. Specifically, since the substrate (i.e., sucrose) in Figure 6a and Figure 7a was adequate, the enzyme activity (5477.0 ± 265.3 U) was higher in Figure 6a than that (4277.0 ± 174.5 U) in Figure 7a. Comparison of the results shown in Figure 5a,c revealed that the cell concentration was lower in Figure 5c, which resulted in more substrate (i.e., sucrose) facilitating FOS biosynthesis. When the substrate concentration was high, the results would be similar to those shown in Figure 8a (i.e., the process in Figure 5). Thus, it may be concluded that the fed-batch culture was suitable for FOS production, especially for the production of FOS with a high polymerization degree. In fact, FOS production has been conducted successfully by Schorsch et al. [18]. Furthermore, it was speculated that the cells were damaged in the membrane system, which resulted in the low FOS production. The next section discusses what happens when the cell activity was maintained in a repeated batch culture and the substrate (i.e., sucrose) was in excess.

#### 2.2.3. FOS Production in a Repeated-Culture with 1.6 L Broth Discharged Each Time

**Figure 9:**
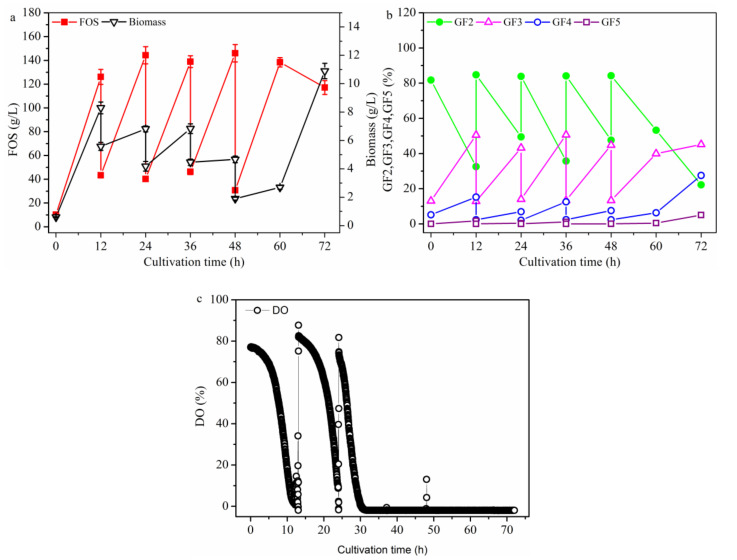
FOS production in a repeated-culture with 1.6 L broth discharged each time. After initial batch fermentation culture for 12 h, about 1.6 L of the broth was removed, and 1.6 L of the fresh medium was fed into the bioreactor. Fermentation was restarted under the same conditions as the batch fermentation in five cycles. The time courses refer to (**a**) FOS and biomass, (**b**) GF2, GF3,GF4 and GF5, and (**c**) DO. The working volume was 1.8 L. Data are given as the mean ± SD, *n* = 2.

β-Fructofuranosidase production was found to be successfully achieved by repeated batch fermentation with immobilized *Aspergillus japonicus* [24]. In addition, a high concentration of the target product was also achieved from biosynthesis using *A. pullulans* with repeated batch culture [36]. Thus, FOS production was conducted using repeated batch culture to determine whether FOS could be effectively biosynthesized from growing cells through direct fermentation production. As shown in Figure 9, a constant concentration (110 g/L) of new FOS was produced in every cycle after culturing for 12 h, but the new increase in cell concentration decreased with increasing cycles. After three cycles, a small increase in new cells was detected. However, in the 5th cycle, no fresh medium was added in the broth after culturing for 12 h, and the test was not stopped to further detect the FOS variation. It was found that after culturing for 12 h, the cells grew fast but FOS decreased faster, indicating that FOS production was not closely associated with cell growth. Therefore, for FOS production using the one-stage process, it was better to control the cell growth to decrease the FOS yield from sucrose. Taken together, under the optimal fermentation condition with the DO concentration being no less than 5% in the repeated-batch culture, maximum FOS concentration of 548.3 ± 37.4 g/L, yield of 68.6% ± 2.6% (g FOS/g sucrose), and productivity of 9.13 ± 0.36 g·L^−1^·h^−1^ were obtained in the repeated-batch culture. Compared with the results reported by Dominguez et al. [3] for *A. pullulans* fermentation using a one-stage process in batch culture, the FOS concentration, yield, and productivity were 4.3 times, 7.2%, and 1.55 times higher, respectively. Compared with the results reported by Muñiz-Márquez et al. [10] for *Aspergillus oryzae* DIA-MF fermentation using aguamiel as the substrate by a one-stage process in batch culture, FOS concentration, yield, and productivity were 26.0 times, 130.0%, and 9.9 times higher, respectively. Compared with the results reported by Nobre et al. [13] for *Penicillium citreonigrum* using a one-stage process in batch culture, the FOS concentration, yield, and productivity were 3.3 times, 6.1%, and 3.0 times higher, respectively. Compared with the results reported by Schorsch et al. [18] for *Aspergillus* sp. fermentation using a one-stage process in fed-batch culture, the FOS concentration, yield, and productivity were 1.9 times, 11.3%, and 44.6% higher, respectively. Therefore, the repeated-batch culture at 25 °C and pH 5.5 under DO being no less than 5%, discharging 500 mL broth at each time, was the best operating mode for FOS production.

## 3. Materials and Methods

### 3.1. Design of Research Works

#### 3.1.1. Effect of DO on FOS Production

First, the effect of stirring speed on FOS production was studied. To investigate the effect of stirring speeds on FOS production, the A. pullulans ipe-3 strain was cultivated in a 2.7-L bioreactor at pH 5.5, aeration rate 1.8 L/min, temperature 25 °C with different stirring speeds (i.e., 400, 600, 800 and 1000 rpm).

Second, under the selected stirring speed, the effect of aeration rates on FOS production was carried out. To investigate the effect of aeration rates on FOS production, the *A. pullulans* ipe-3 strain was cultivated in a 2.7-L bioreactor at pH 5.5 and temperature 25 °C with different aeration rates (i.e., 0.9, 1.8 and 2.7 L/min).

Third, under the selected stirring speed and aeration rate, the effect of partial pressures of the bioreactor on FOS production was carried out. To investigate the effect of partial pressures of the bioreactor on FOS production, the *A. pullulans* ipe-3 strain was cultivated in a 2.7 L bioreactor at pH 5.5, aeration rate 1.8 L/min, and temperature 25 °C with different pressures of the fermenter (i.e., 0, 0.4 and 0.8 bar). The different pressures were achieved by adjusting the valve opening in the exhaust pipe of the fermenter. When studying the effect of partial pressure of the bioreactor, the gauge pressure was used. Especially, the 0 bar means the gauge pressure at 1 atmospheric pressure.

Fourth, under the selected stirring speed, aeration rate and partial pressures of the bioreactor, the effect of temperature on FOS production was carried out. To investigate the effect of fermentation temperatures on FOS production, the *A. pullulans* ipe-3 strain was cultivated in a 2.7-L bioreactor at pH 5.5, aeration rate 1.8 L/min, and stirring speed 600 rpm with different fermentation temperatures of the fermenter (i.e., 25, 30 and 35 °C).

#### 3.1.2. Effect of Fermentation Mode on FOS Production

First, to further detect DO on FOS production, the repeated-batch culture, discharging 500 mL broth each time, was carried out. These repeated-batch cultivations at 25 °C and pH 5.5 were, respectively, carried out under 600 rpm and 1.8 L/min (a), 5% DO that was cascaded to stirring speed through Proportion Integral Differential (PID) control (b), and 600 rpm and 0 L/min after culturing under 600 rpm and 1.8 L/min for 12 h (c). For 5% DO control, the DO was allowed to automatically drop to 5% and then controlled constantly by manually adjusting the partial pressure of the bioreactor and automatically adjusting the stirring speed using the PID controller. The fed-solution was 720 g/L sucrose, and the culture was fed at 12, 24, 36, and 48 h after discharging 500 mL of broth each time.

Second, to further detect cell concentration on FOS production in repeated-batch culture, FOS production in a membrane integrated repeated batch culture was carried out. The culture conditions were the same as those in the repeated-batch culture with 500 mL broth discharged each time. The main difference was that no cells remained in the 500 mL of discharged broth. The cells in the discharged broth were recovered using a 300 kDa CéRAM INSIDE tubular ceramic module membrane with an effective surface area of 0.16 m^2^. The cells were then recycled and passed into the bioreactor. The schematic of the membrane system is shown in Figure S1.

Third, to further detect the enzyme dosage and temperature on FOS production, the effect of (a) adding enzyme and (b) temperature on FOS production was carried out. Enzyme indicates the fermentation broth that was used as the crude enzyme. The conditions were the same as in the enzyme activity measurement experiment but the amount of broth added was varied in (a), while the temperature was varied in (b).

Fourth, to determine if FOS could be effectively biosynthesized from growing cells through direct fermentation production, FOS production was conducted using another repeated batch culture. Under this fermentation mode, the initial fermentation batch was cultured for 12 h after FOS production using repeated batch culture, 1.6 L broth was removed, and 1.6 L fresh medium was then fed into the bioreactor. Fermentation was restarted under the same conditions as batch fermentation.

### 3.2. Microorganism

*Aureobasidium pullulans* ipe-3 (accession number KY618121) was used in this study. The organism was stored at the State Key Laboratory of Biochemical Engineering, Institute of Process Engineering, Chinese Academy of Sciences, Beijing, China. The strain was maintained on Czapek Dox agar slants (Beijing Aoboxing Bio-tech Co., Ltd., Beijing, China) at 4 °C.

### 3.3. Culture Medium

The compositions (g/L in deionized water) of the seed and fermentation media were as follows: sucrose 200, yeast extract 10, NaNO_3_ 5, KH_2_PO_4_ 4, KCl 0.5, FeSO_4_·7H_2_O 0.01, K_2_SO_4_ 0.35 and MgSO_4_·7H_2_O 0.5.

### 3.4. Culture Method

In all of the fermentation experiments, the seed culture of the ipe-3 strain was prepared by inoculating a loop full of mycelia from 5-day-old cells into 500-mL Erlenmeyer flasks containing 100 mL seed medium and then incubating at 25 °C for 2 days in a rotary shaker (HYG-A, Taicang Experimental Equipment Factory, Taicang, China) at 150 rpm. The seeds prepared for the bioreactor were cultured in four 500 mL Erlenmeyer flasks each time. After culturing and mixing, the seed culture broth (200 mL) was transferred into a 2.7 L bioreactor (BioFio^®^ 110, New Brunswick Scientific, San Francisco, USA) containing 1.6 L of medium. The compositions (g/L) of the 1.6-L media were as follows: sucrose 225, yeast extract 11.25, NaNO_3_ 5.625, KH_2_PO_4_ 4.5, KCl 0.5625, FeSO_4_·7H_2_O 0.01125, K_2_SO_4_ 0.39375, and MgSO_4_·7H_2_O 0.5625 in deionized water. The pH of the medium was maintained at 5.5 by the automatic addition of 2 M NaOH or 1 M H_2_SO_4_. The DO concentration was detected using an on-line DO probe (P52201018, S8238050, Mettler Toledo, Zurich, Switzerland) and the DO electrode was calibrated according to the procedure described by Cao et al. [21] (Supplementary Information). The pO_2_ in the saturated sodium sulfite solution was calibrated as zero, while the maximum O_2_ saturation value in the sterilized broth before inoculation under 10 L/min at a stirring speed of 1000 rpm was calibrated as 100% of the DO probe.

### 3.5. Analytical Methods

The culture broth (3 mL) was centrifuged using a high-speed centrifuge (4–16 K, Sigma, Osterode am Harz, Germany) at 10,000 *g* for 10 min, and the resulting supernatants were used to determine the concentrations of FOS, sucrose, fructose and glucose. The FOS were purchased from Shanghai Acmec Biochemical Co. Ltd., Shanghai, China. Sucrose, fructose and glucose were purchased from Sinopharm Chemical Reagent Co., Ltd., Beijing, China. To measure the biomass, cells were washed three times with 6 mL distilled water and then dried to constant weight at 90 °C. The concentrations of the sugars were obtained using a high-performance liquid chromatography (HPLC) apparatus (LC-20AT, Shimadzu, Kyoto, Japan) equipped with an reflective index (RI)-detector using the Asahipak NH2P-50 4E column (Shodex, Tokyo, Japan). The column temperature was set to 30 °C, and a mixture of acetonitrile/distilled water (7:3, *v*/*v*) was applied as the mobile phase at a flow rate of 1 mL/min. The enzyme activity was measured as described by Shin et al. [29] (refer to the Supplementary Information for the details describing the enzyme assay). Statistical analysis of the different experimental groups was conducted by subjecting the experimental data to one-way analysis of variance (ANOVA) using the OriginPro 2018 software (Origin Lab Corporation, Northampton, MA, USA) at a 95% confidence level. The data presented in Figure 1, Figure 2, Figure 3, Figure 4, Figure 5, Figure 6, Figure 7, Figure 8 and Figure 9 are the average values with error bars.

The FOS yield (*Y_p__/s_*), specific FOS production (FOS/biomass)) (*Y_p__/b_*), specific activity of fructosyltransferase (fructosyltransferase activity/biomass) (*Y_e__/b_*), and FOS productivity are expressed as follows:

$$Y_{p/s}=\frac{\mathrm{final}\;\mathrm{FOS}\;\mathrm{concentration}}{\mathrm{initial}\;\mathrm{sucrose}\;\mathrm{concentration}\;–\mathrm{residual}\;\mathrm{sucrose}\;\mathrm{concentration}}$$

$$Y_{p/b}=\frac{\mathrm{final}\;\mathrm{FOS}\;\mathrm{concentration}}{\mathrm{final}\;\mathrm{biomass}\;\mathrm{concentration}\;-\mathrm{initial}\;\mathrm{biomass}\;\mathrm{concentration}\;}$$

$$Y_{e/b}=\frac{\mathrm{final}\;\mathrm{enzyme}\;\mathrm{activity}-\mathrm{initial}\;\mathrm{enzyme}\;\mathrm{activity}\;}{\mathrm{final}\;\mathrm{biomass}\;\mathrm{concentration}\;-\mathrm{initial}\;\mathrm{Biomass}\;\mathrm{concentration}\;}$$

$$\mathrm{FOS}\;\mathrm{productivity}\;=\frac{\mathrm{final}\;\mathrm{FOS}\;\mathrm{concentration}\;}{\mathrm{cultivation}\;\mathrm{time}}$$

where the unit of sugar concentration, FOS concentration or biomass concentration is g/L; the unit of fructosyltransferase activity is U; the unit of cultivation time is hour (h).

## 4. Conclusions

In this study, the effects of DO concentration and fermentation mode on FOS using *A. pullulans* were investigated. It was found that the DO value could not be improved by varying only one of the factors (i.e., stirring speed, aeration rate, or partial pressure of the bioreactor). Moreover, cell growth and enzyme production were found to be enhanced at a high DO value, while the high cell concentration and enzyme production decreased FOS production. With the help of different fermentation modes, it was found that the DO affected FOS production and the composition of FOS with different polymerization degrees, and FOS production was not closely associated with cell growth. In addition, the repeated-batch culture was suitable for producing FOS with a high polymerization degree. Under the optimal fermentation condition, a maximum FOS concentration of 548.3 ± 37.4 g/L, yield of 68.6% ± 2.6% (g FOS/g sucrose), and productivity of 9.13 ± 0.36 g·L^−1^·h^−1^ were obtained. Therefore, the results of the present study not only offer an effective strategy to improve FOS production, but also provide a good reference for the process development and optimization of FOS with a high polymerization degree.

## Data Availability

The data presented in this study are available on request from the corresponding author.

## Supplementary Materials

The following are available online, Figure S1: Schematic diagram of the membrane system for FOS production in a membrane bioreactor.

## Abbreviations

FOS	fructo-oligosaccharides
DO	dissolved oxygen
GF4	1-fructofuranosyl nystose
GF5	1,1,1,1-kestohexose
GF2	1-kestose
GF3	nystose
Y_p__/s_	FOS yield
Y_p__/b_	specific FOS production (FOS/biomass),
Y_e__/b_	specific activity of fructosyltransferase (fructosyltransferase activity/biomass)
