# Supplementary material for: Fermentative Production of Fructo-Oligosaccharides Using Aureobasidium pullulans: Effect of Dissolved Oxygen Concentration and Fermentation Mode
Source: Molecules. 2021 Jun 24;26(13):3867. doi: 10.3390/molecules26133867 (PMC8270319; doi:10.3390/molecules26133867)
Supplement: Supplementary file 1 [file molecules-26-03867-s001.zip › molecules-1255092 supplementary conversion.pdf]

molecules

Supplementary Materials

Fermentative Production of Fructo-Oligosaccharides Using *Aureobasidium Pullulans*: Effect of Dissolved Oxygen Concentration and Fermentation Mode

Xinquan Liang [1,2], Chenglin Li [1,2], Weifeng Cao [2,3,*], Weilei Cao [2,3], Fei Shen [2,3] and Yinhua Wan [2,3]

[1] Department of Sugar Engineering, College of Light Industry and Food Engineering, Guangxi University, Nanning 530004, China; lxq10713@163.com (X.L.); a860835337@gmail.com (C.L.)
[2] State Key Laboratory of Biochemical Engineering, Institute of Process Engineering, Chinese Academy of Sciences, Beijing 100190, China; lxq10713@163.com (X.L.); a860835337@gmail.com (C.L.); wfcao@ipe.ac.cn (W.C.); wlcao@foxmol.com (W.C.); fshen@ipe.ac.cn (F.S.); yhwan@ipe.ac.cn (Y.W.)
[3] School of Chemical Engineering, University of Chinese Academy of Sciences, Beijing 100049, China; wfcao@ipe.ac.cn (W.C.); wlcao@foxmol.com (W.C.); fshen@ipe.ac.cn (F.S.); yhwan@ipe.ac.cn (Y.W.)
* Correspondence: wfcao@ipe.ac.cn; Tel./Fax: +86-10-62650673

Citation: Liang, X.; Li, C.; Cao, W.; Cao, W.; Shen, F.; Wan, Y. Fermentative Production of Fructo-Oligosaccharides Using *Aureobasidium pullulans*: Effect of Dissolved Oxygen Concentration and Fermentation Mode. *Molecules* **2021**, *26*, 3867. https://doi.org/10.3390/molecules26133867

Academic Editor: Yasuhiro Ozeki

Received: 26 May 2021
Accepted: 22 June 2021
Published: 24 June 2021

Publisher's Note: MDPI stays neutral with regard to jurisdictional claims in published maps and institutional affiliations.

Copyright: © 2021 by the authors. Licensee MDPI, Basel, Switzerland. This article is an open access article distributed under the terms and conditions of the Creative Commons Attribution (CC BY) license (http://creativecommons.org/licenses/by/4.0/).

1. Enzyme Assay

During the cultivation, 5 mL of the culture broth was centrifuged using a table-top centrifuge (5000 rpm) for 10 min at room temperature and the supernatant was collected for the determination of extracellular enzyme activity. The cells centrifuged from the 5 mL culture broth were washed and resuspended up to 5 mL with saline and then used for the determination of intracellular enzyme activity. One unit (1 U) defined as the amount of enzyme activity required to produce 1 μmol of glucose/min under the following conditions: (a) pH 5.5, (b) temperature 55 °C, (c) reaction time 1 h, (d) reaction mixture consisting of the following composition: 7.5 mL of sucrose 800 g/L, 2.3 mL of 0.1 M citrate buffer (pH 5.5), and 0.2 ml enzyme sample. The enzyme reaction was stopped by heating at 100 °C for 15 min and the released glucose from the enzyme reaction was measured by by a HPLC apparatus (LC20AT, Shimadzu, Japan) equipped with a RI-detector using the Asahipak NH2P-50 4E column (Shodex, Japan).

2. The Procedure for DO Electrode Calibration

Briefly, the procedure for DO electrode calibration involved reducing the pO2 in the saturated sodium sulfite solution to zero and then following the increase in pO2 over time after the resumption of aeration and stirring speed, until maximum O_2 saturation was reached in the sterilized broth before inoculation under pH 5.5, 25°C and air saturation at 1 atm. The maximum O_2 saturation value, 8.9 mg L^{-1} achieved at aeration rate 10 $Lmin^{-1}$ and stirring speed 1000 rpm, was calibrated as 100% of the DO probe. Then DO value detected by the DO probe in the fermentation process was the relative value compared with the calibration.

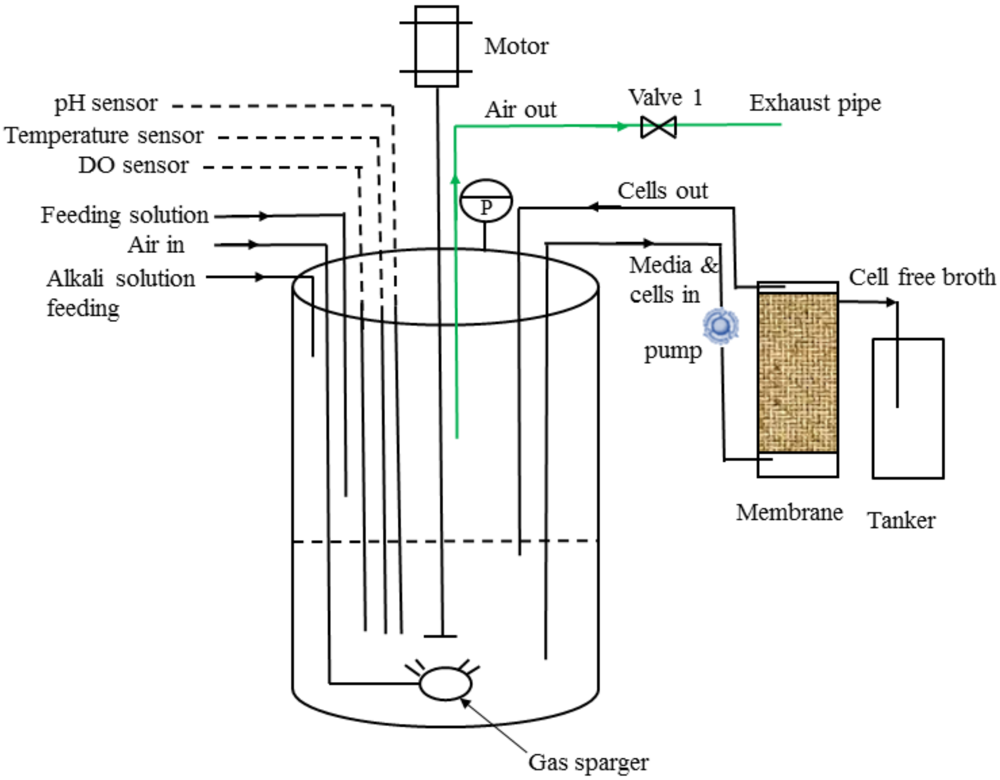

Figure S1. Schematic diagram of the membrane system for FOS production in a membrane bioreactor.
